# The Molecular and Spatial Epidemiology of Typhoid Fever in Rural Cambodia

**DOI:** 10.1371/journal.pntd.0004785

**Published:** 2016-06-22

**Authors:** Duy Pham Thanh, Corinne N. Thompson, Maia A Rabaa, Soeng Sona, Sun Sopheary, Varun Kumar, Catrin Moore, Nga Tran Vu Thieu, Lalith Wijedoru, Kathryn E. Holt, Vanessa Wong, Derek Pickard, Guy E. Thwaites, Nicholas Day, Gordon Dougan, Paul Turner, Christopher M. Parry, Stephen Baker

**Affiliations:** 1 The Hospital for Tropical Diseases, Wellcome Trust Major Overseas Programme, Oxford University Clinical Research Unit, Ho Chi Minh City, Vietnam; 2 Centre for Tropical Medicine, Oxford University, Oxford, United Kingdom; 3 The London School of Hygiene and Tropical Medicine, London, United Kingdom; 4 Cambodia-Oxford Medical Research Unit, Angkor Hospital for Children, Siem Reap, Cambodia; 5 Department of Biochemistry and Molecular Biology, Bio21 Molecular Science and Biotechnology Institute, University of Melbourne, Parkville, Victoria, Australia; 6 The Wellcome Trust Sanger Institute, Hinxton, Cambridgeshire, United Kingdom; 7 The Department of Medicine, University of Cambridge, Cambridge, United Kingdom; 8 School of Tropical Medicine and Global Health, Nagasaki University, Nagasaki, Japan; Massachusetts General Hospital, UNITED STATES

## Abstract

Typhoid fever, caused by the bacterium *Salmonella* Typhi, is an endemic cause of febrile disease in Cambodia. The aim of this study was to better understand the epidemiology of pediatric typhoid fever in Cambodia. We accessed routine blood culture data from Angkor Hospital for Children (AHC) in Siem Reap province between 2007 and 2014, and performed whole genome sequencing (WGS) on the isolated bacteria to characterize the *S*. Typhi population. The resulting phylogenetic information was combined with conventional epidemiological approaches to investigate the spatiotemporal distribution of *S*. Typhi and population-level risk factors for reported disease. During the study period, there were 262 cases of typhoid within a 100 km radius of AHC, with a median patient age of 8.2 years (IQR: 5.1–11.5 years). The majority of infections occurred during the rainy season, and commune incidences as high as 11.36/1,000 in children aged <15 years were observed over the study period. A population-based risk factor analysis found that access to water within households and increasing distance from Tonle Sap Lake were protective. Spatial mapping and WGS provided additional resolution for these findings, and confirmed that proximity to the lake was associated with discrete spatiotemporal disease clusters. We confirmed the dominance of MDR H58 *S*. Typhi in this population, and found substantial evidence of diversification (at least seven sublineages) within this single lineage. We conclude that there is a substantial burden of pediatric typhoid fever in rural communes in Cambodia. Our data provide a platform for additional population-based typhoid fever studies in this location, and suggest that this would be a suitable setting in which to introduce a school-based vaccination programme with Vi conjugate vaccines.

## Author Summary

Typhoid fever is an infectious disease caused by the bacterium *Salmonella* Typhi. The disease is generally restricted to those living in low-income settings with poor sanitation. Typhoid fever is a common cause of fever requiring hospital treatment in Cambodia, but limited data is available on the epidemiology of the disease. To better understand typhoid fever in Cambodia, we accessed routine hospital data for typhoid fever from a single healthcare facility treating sick children in Siem Reap in the central Cambodia between 2007 and 2014. We mapped the location of these cases and examined population-based risk factors for reported disease. Additionally, we decoded the genomes of the *S*. Typhi isolated from children attending the hospital to understand how the organism has evolved and spread throughout the population. We found a large burden of typhoid fever in children in this largely rural setting in central Cambodia. We also found that disease was associated with the rainy season and that living close to Tonle Sap Lake increased the risk of disease. The genomes of the sequenced bacteria showed that a diverse range of strains were circulating during the study, and allowed us to identify signatures of location- and time-specific outbreaks. Our work provides baseline data for additional typhoid fever studies in the population living in this location and findings suggest that rural Cambodia would be a suitable setting in which to introduce a school-based vaccination program with new typhoid vaccines.

## Introduction

The bacterium *Salmonella enterica* serovar Typhi (*S*. Typhi) is the cause of the human infection typhoid fever, a systemic disease predominantly diagnosed in children and young adults in low-income settings [1]. *S*. Typhi is primarily contracted via ingestion of food or water contaminated with human feces from patients excreting the organism, and typhoid fever remains a major public health issue in areas with poor sanitation and limited access to safe water [2]. The control of typhoid fever is largely dependent on improving the availability of clean water, hygienic food preparation and access to adequate sanitation, but such interventions are substantial challenges in many locations where typhoid remains endemic [3]. As a result, active case detection and appropriate antimicrobial therapy are currently the principal methods for controlling this disease in endemic locations. The lack of rapid and reliable diagnostics and the emergence of antimicrobial resistance (AMR) reduce the effectiveness of these strategies [4,5].

The World Health Organization (WHO) currently recommends the use of licensed typhoid vaccines in areas where the burden of typhoid fever is high and AMR organisms are prevalent, though limited programmatic use of vaccines has occurred in endemic countries [6]. Identifying areas that have a burden of typhoid fever warranting immunization programmes can be challenging. Much of the current focus for vaccine implementation is on highly populated urban slums with poor infrastructure [7–9]. However, it is unclear if the epidemiology of typhoid is similar between urban and rural regions in the developing world. Understanding the dominant modes of transmission and epidemiological risk factors for typhoid fever in both urban and rural endemic regions is vital for controlling and preventing the disease [3].

New molecular tools now permit an unprecedented insight into how *S*. Typhi may be circulating locally and internationally [10,11,7]. Comparing the composition of phylogenetically informative Single Nucleotide Polymorphisms (SNPs) across the *S*. Typhi genome allows the subtyping of populations of *S*. Typhi and inference of evolutionary relationships between isolates [12,13]. SNP-based typing methods, and now whole genome sequencing (WGS), have been successfully used to study the molecular epidemiology of typhoid in different settings, revealing the importance of environmental transmission and the diversity of commonly co-circulating haplotypes of *S*. Typhi within localized human populations [7,14–16]. Such molecular approaches play an important role in identifying dominant transmission pathways and can also capture both the emergence of AMR and the dynamics of the bacterial population. We now know that the current population of *S*. Typhi has been driven by a clonal expansion and international dispersal of a specific haplotype (H58) in Asia and Africa. This H58 haplotype now dominates internationally and is associated with a multidrug resistant (MDR) phenotype (non-susceptibility to ampicillin, chloramphenicol and trimethoprim-sulphamethoxazole) and reduced susceptibility to fluoroquinolones [11].

Typhoid fever is endemic in Cambodia, although only limited data regarding the morbidity, mortality and risk factors for the disease are available in published literature. A community-based study conducted near Phnom Penh (the capital city) between 2006 and 2009 reported routine isolation of MDR *S*. Typhi from the blood of febrile patients [17] Additionally, a hospital-based cross-sectional study of pediatric bloodstream infections in a children’s hospital in Siem Reap (in northwest Cambodia, near the World Heritage Site of Angkor Wat) between 2007 and 2011 found that *S*. Typhi was the most commonly isolated pathogen in this setting and confirmed the presence and dominance of H58 *S*. Typhi (98/102; 96% of *S*. Typhi isolates) exhibiting reduced susceptibility to ciprofloxacin [18,19]. In the present study, we aimed to utilize the precision of WGS to characterize the H58 *S*. Typhi population in Siem Reap, Cambodia. Further, we combined the resulting phylogenetic information with additional epidemiological approaches to investigate the spatiotemporal distribution of *S*. Typhi and population-level risk factors for typhoid fever infection in this location.

## Methods

### Ethics statement

The study involved characterization of stored bacterial isolates cultured from specimens taken for routine clinical care. Therefore, it was not possible to obtain consent from the patient or their parent/guardian for participation in this retrospective study, but all patient data was anonymized. The study protocol was reviewed and approved by both the Angkor Hospital for Children Institutional Review Board (AHC IRB; reference 423/13) and the Oxford Tropical Research Ethics Committee (OxTREC; reference 512–13).

### Study site and setting

This study was conducted at Angkor Hospital for Children (AHC) in Siem Reap City in Cambodia between January 2007 and December 2014. AHC is one of two pediatric hospitals in Siem Reap City and has approximately 125,000 attendees and 4,000 admissions per year. The patients attending AHC are <16 years of age and come from a wide geographical radius and attend the hospital for various conditions. The majority of patients reside in the province of Siem Reap, which is located in northwest Cambodia and is bordered in the south by the Tonle Sap Lake, the largest freshwater lake in Southeast Asia. According to available census data, the province had a population of 896,443 people living in an area of 10,299 km^2^ in 2008; the province is subdivided administratively into 12 districts, 100 communes (which are within districts) and 907 villages. [20]. Cambodia has a tropical climate with a dry and wet season each year. During the wet season (April–October) the area of the Tonle Sap Lake can expand dramatically, increasing from 3,500 km^2^ up to approximately 14,500 km^2^, with the depth increasing from 0.5 m up to 6–9 m [21].

### Definition of the case population and the control population

Case and control populations were identified from the electronic hospital and laboratory information system of AHC. For the purposes of this study, the case population was defined as the population of hospital inpatients from whom *S*. Typhi was isolated from a blood culture. The control population was defined as the patient population admitted to AHC who did not have typhoid fever based on the recorded discharge diagnosis (International Classification of Disease (ICD)-10 code). Patients with a discharge diagnosis of typhoid fever but without blood culture confirmation (n = 410) were not included in the risk factor analysis. Additionally, for the mapping and population risk factor analyses, cases that lived outside of a 100km radius from AHC were excluded. Data on age, sex, home location (commune level), admission and discharge dates for cases and controls were extracted from the electronic hospital information system. If a case or control was readmitted to the hospital with the same discharge diagnosis within a seven-day period, only the initial admission was included in the analysis.

### Data sources

Commune-level census data were obtained from the Cambodian National Report on General Population Census of 2008 [20]. The extracted information included details regarding demographic indicators, age structure, literacy and education, housing and household characteristics, and access to toilet facilities and drinking water. Based on this report, a commune was classified as urban if the population density exceeded 200/km^2^, less than half of men were employed in agriculture and the total population exceeded 2,000. Monthly average precipitation was collected from Siem Reap Weather Station and MRCS (Mekong River Commission Secretariat) [22]. Shuttle Radar Topography Mission (SRTM) elevation data were obtained from the CGIAR Consortium for Spatial Information (CGIAR-CSI) [23]. Shapefile layers containing 2008 commune-level population census data were accessed from Open Development Cambodia, an open-access data website providing data on Cambodia and its economic and social development (http://www.opendevelopmentcambodia.net).

### Typhoid diagnosis and bacterial identification

Routine diagnosis of typhoid fever was performed by blood culture. Blood (1–4 ml) was taken for bacterial culture from all patients with fever including those with a clinical suspicion of typhoid fever. Blood was inoculated into media containing tryptone soya broth and sodium polyanethol sulphonate, up to a total volume of 25mL. Blood culture bottles were incubated for up to seven days, with blind sub-cultures at 24 hours, 48 hours and 7 days or if the broth was cloudy. Positive bottles were subcultured onto sheep blood, chocolate and MacConkey agar and presumptive *Salmonella* colonies were identified using standard biochemical tests and serotype-specific antisera (Murex Biotech, Dartford, England). Antimicrobial susceptibility testing was performed by the modified Bauer-Kirby disc diffusion method with zone size interpretation based on CLSI guidelines [24]. Etests were used to determine MICs, following the manufacturer's recommendations (bioMérieux, France). Ciprofloxacin MICs were used to categorize *S*. Typhi isolates as susceptible (≤0.06 μg/mL), intermediate (0.12–0.5 μg/mL) and resistant (≥1 μg/mL) following CLSI guidelines [24].

### Statistical analysis

Rates of hospitalized typhoid fever were calculated at the commune level using the population under the age of 15 years from 2008. Multivariable negative binomial regression was used to identify commune-level risk factors associated with the rate of cases per 1,000 population under the age of 15 years. Interaction between commune level factors was evaluated using the likelihood ratio test. Variables included in the evaluation of the final model included those with significant associations (*p*<0.10) in the univariate analysis and *a priori* sanitation and water source variables. Variables that did not add significantly to the fit of the final model (determined by the likelihood ratio test) were not included. All analyses were performed in STATA (v13, College Station, TX, USA) and plots were created in R v3.1.1 (R Foundation for Statistical Computing, Vienna, Austria, https://cran.r-project.org/) using ggplot2 [25].

### Spatiotemporal clustering detection

Spatiotemporal clustering analysis was performed using Moran’s I and SaTScan methodologies. First, Moran’s I test was used to evaluate global autocorrelation amongst communes that reported at least one case (n = 78) of typhoid fever in GeoDa software (v1.6.7, https://geodacenter.asu.edu/). This test statistic provides an evaluation of whether the rates across the area of interest are spatially random (Moran’s I = 0), over-dispersed (Moran’s I<0) or clustered (Moran’s I>0) [26]. Next, Kulldorff’s scan statistic in SaTScan (v9.1.1, http://www.satscan.org/) was used to identify the location of clusters of communes with high rates of typhoid fever over space and time [27,28]. A cylindrical window was used to scan the area for clusters, with the size of the circle corresponding to the spatial scan and the height of the cylinder corresponding to time. The significance of the detected clusters was assessed by a likelihood ratio test, with a *p*-value obtained by 999 Monte Carlo simulations generated under the null hypothesis of random spatiotemporal distribution. In this analysis, scan windows were used to fit discrete Poisson models. For the sublineage-specific analyses, all case communes were included and those without cases of a specific sublineage were classified as having 0 cases. The upper limit for cluster detection was specified as 25% of the study population over each year. All maps were created in ArcGIS 10.2 (ESRI, Redlands, CA, USA).

### Whole-genome sequencing and phylogenetic analysis

Of the 284 *S*. Typhi isolates collected between 2007 and 2014, a total of 209 (74%) collected between 2007 and 2012, were subjected to genomic DNA extraction using the Wizard Genomic DNA Extraction Kit (Promega, Wisconsin, USA) (S1 Table). Two micrograms of genomic DNA was subjected to WGS on an Illumina HiSeq2000 platform following the manufacturer’s recommendations to generate 100bp paired-end reads. All reads were mapped to the reference sequence of *S*. Typhi strain CT18 (Accession no: AL513382), plasmid pHCM1 (AL513383) and pHCM2 (AL513384) using SMALT (version 0.7.4). Candidate SNPs were called against the reference sequence using SAMtools and filtered with a minimal mapping quality of 30 and a quality ratio cut-off of 0.75. The allele at each locus in each isolate was determined by reference to the consensus base in that genome, using *samtools mpileup* and removing low confidence alleles with consensus base quality ≤20, read depth ≤5 or a heterozygous base call. SNPs in phage regions, repetitive sequences or recombinant regions were excluded, resulting in a final set of 750 chromosomal SNPs. Strains belonging to haplotype H58 were defined by the SNP *glpA*-C1047T (position 2,348,902 in *S*. Typhi CT18, BiP33, as previously described [12,13]).

A maximum likelihood phylogenetic tree was constructed from a 188 chromosomal SNP alignment of H58 isolates with RAxML (version 7.8.6) using the generalized time-reversible model (GTR) and a gamma distribution to model site-specific rate variation (GTR+Г nucleotide substitution model in RAxML). Support for the ML phylogeny was assessed via 1,000 bootstrap pseudo-analyses of the alignment data. Phylogenetic subgrouping was defined based on monophyletic groups (lineages) with well-supported bootstrap value (≥85%).

To investigate the short-term divergence within the bacterial population and the transmission within the local population, a minimum spanning tree was reconstructed from the SNP alignment of lineage III and lineage IV identified in the ML tree (accounting for 95% of isolates) using the goeBURST algorithm in Phyloviz software (version 1.1) [29]. This algorithm identified seven sublineages based on similarity among allelic profiles and frequency of isolation within the population. Sequences with identical SNP profiles and isolated at the highest frequency within each sublineage were assigned as founder genotypes (viewed as the central nodes within each of the sublineages), with descendant genotypes (represented by terminal nodes surrounding the founder genotype) assigned based on similarity to founder SNP profiles. These descendent genotypes can differ from the parental genotype by a single or multiple SNPs. The raw sequence data for this study are available in the European Nucleotide Archive (ENA) under the accession numbers described in S1 Table.

## Results

### Baseline characteristics

Between 2007 and 2014, there were 284 microbiologically confirmed cases of typhoid fever caused by *S*. Typhi at AHC in Siem Reap. *S*. Paratyphi A was uncommon, with only three cases in 2008 followed by an isolated outbreak in 2013–2014 (38 cases). A total of 262/284 (93%) of the confirmed *S*. Typhi cases lived within a 100 km radius of AHC and spanned 78 communes; these 78 communes were selected for the spatial comparison and the typhoid fever population level risk factor analyses. During this same period there were 19,877 admissions with an ICD-10 discharge diagnosis other than typhoid fever originating from the same geographic area. The baseline characteristics of all communes and those with at least one case of typhoid fever are shown in Table 1.

**Table 1:** Baseline characteristics of all communes and those with at least one case of typhoid fever.

Characteristic	All communes		Typhoid communes	
	median	IQR	median	IQR
	n = 243		n = 78	
Population density/km^2^	105.7	53–210	119.4	60–214
Elevation, m	17	12–28	18	11–35
Distance to lake, km	45.3	24–63	30.0	14–49
Average household size	4.8	4.6–5.0	5.0	4.8–5.1
Percent of population <15 yr	36.4%	34–39%	36.9%	35–39%
Median age of population, yr	19.5	18–21	19.4	18–20
Adult literacy	72.8%	59–82%	69.3%	57–77%
Female adult literacy	65.3%	50–75%	62.5%	49–71%
Total attending school	28.6%	26–31%	28.0%	24–31%
Female attending school	26.8%	24–29%	26.0%	23–28%
Female education >25 years /1,000 population				
Primary not completed	85.6	63–101	78.5	59–101
Primary/Lower secondary	27.8	16–55	22.0	16–39
Secondary or above	0.51	0.1–1.6	0.4	0.1–1.4
Toilet, % of households				
None	83.1%	63–92%	85.2%	68–92%
Sewage	5.3%	2–14%	4.6%	2–15%
Septic tank	3.9%	1–16%	2.8%	1–11%
Pit latrine	2.0%	1–5%	1.2%	1–5%
Drinking water, % of households				
Piped	1.5%	1–4%	2.1%	1–4%
Tube/pipe well	10.2%	3–28%	23.6%	8–63%
Dug well	26.9%	11–56%	23.6%	9–63%
Spring/river	23.9%	4–54%	4.7%	1–27%
Drinking water location, % of households				
Within premises	19.3%	10–35%	27.8%	18–56%
Near premises	31.1%	22–40%	28.0%	21–34%
Away premises	41.8%	23–56%	35.0%	11–52%

IQR: interquartile range

**Fig 1:**
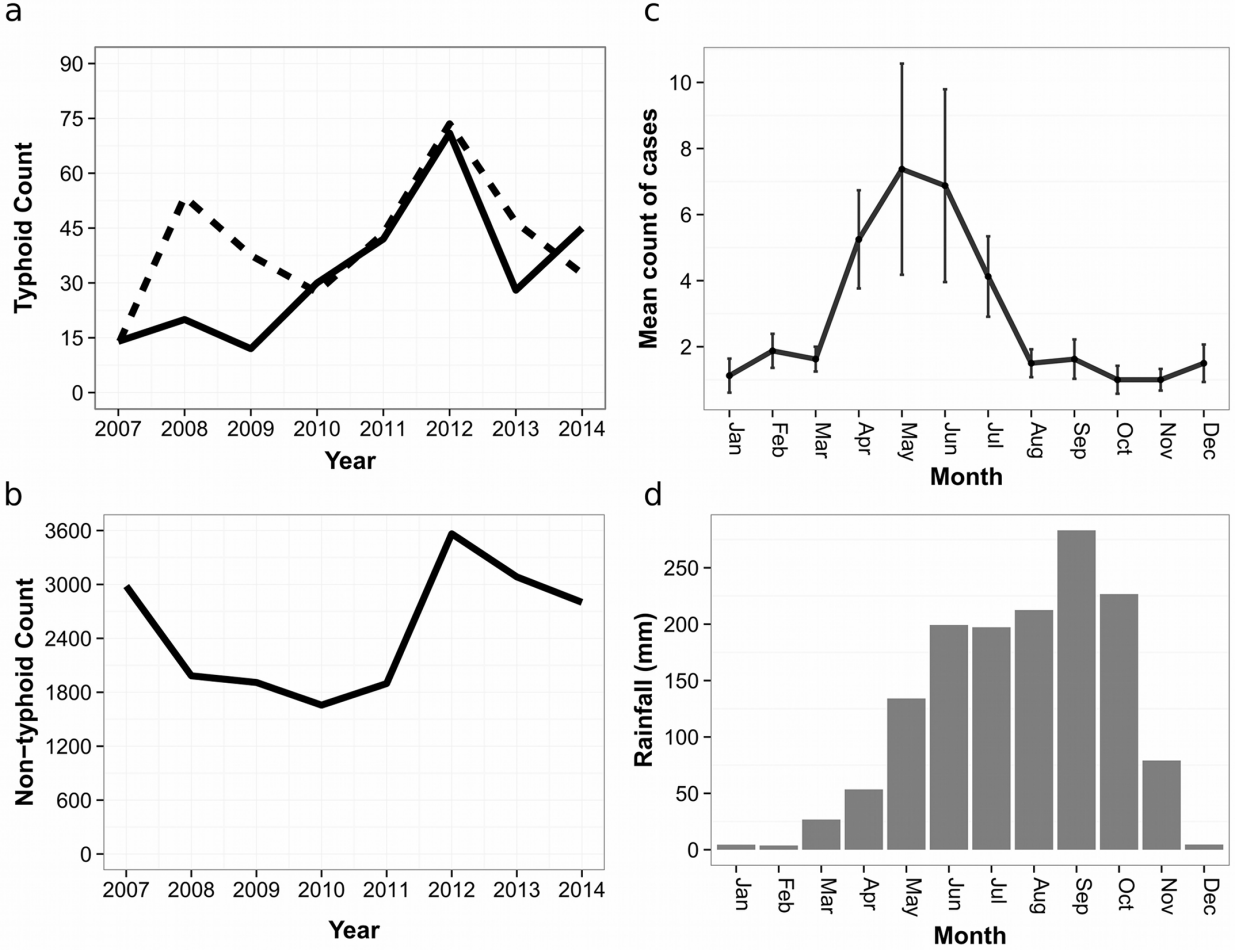
The annual and seasonal distribution of typhoid fever cases at Angkor Hospital for Children in Cambodia. a) The annual number of culture confirmed (solid line) and non-confirmed (broken line) typhoid cases at AHC from 2007 to 2014. b) The annual number of total admissions at AHC from 2007 to 2014. c) The mean monthly count of typhoid cases aggregated from 2007 to 2014. d) The average monthly rainfall (mm) per month over the study period.

Of the 262 cases of typhoid fever living within a 100 km radius of AHC, the median age was 8.2 years (interquartile range (IQR): 5.1–11.5 years). Additionally, 62/262 (24%) of the cases were less than five years of age and 142/262 (54%) were female. As shown in Fig 1a, the absolute number of confirmed cases of typhoid fever increased dramatically (from 12 cases per year to 71 cases per year) between 2009 and 2012, but then declined in 2013 and 2014 (28 and 45 cases in 2013 and 2014; respectively); data from our non-confirmed typhoid cases also reflected this trend. Over this same time period (2009 to 2014) the number of patients attending AHC for other conditions (control population) mirrored the distribution of the cases (Fig 1b). There was seasonal variation in the number of typhoid cases, with the majority of the cases (178/262; 68%) occurring during the early monsoon months (April, May, June and July) (Fig 1c & 1d). In late monsoon months (August to October), the number of cases declined to less than two cases per month and generally remained below this threshold in the dry season (November to March) (Fig 1c & 1d).

### Spatiotemporal clustering of typhoid fever cases

**Fig 2:**
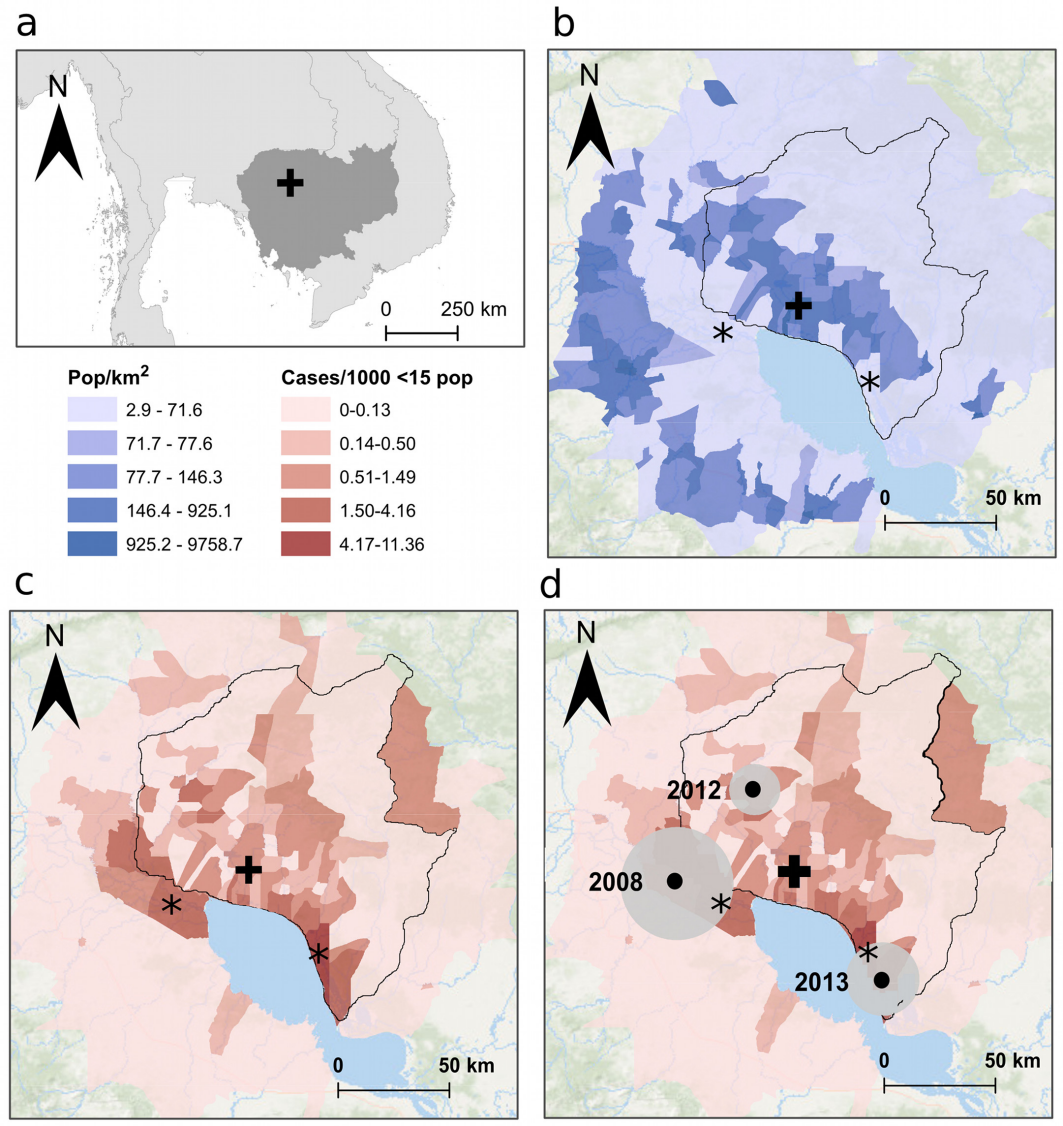
The spatial distribution of typhoid fever cases in Siem Reap province, Cambodia. a) North oriented map of Cambodia, the black cross shows the location of AHC. b) Map showing the population density (people/km^2^, color-coding in key) of the 78 communes within the typhoid study area. AHC is shown by the black cross, the black border denotes Siem Reap province and the left and right asterisks are mark the locations of the communes with highest incidence of typhoid fever; Kaoh Chiveang and Kampong Kleang, respectively. c) Map of the study area showing the rate of reported typhoid cases per 1,000 population under the age of 15 years (color-coding in key). d) Map of the study area showing significant spatiotemporal clusters of typhoid during the study period, the size of the grey circles corresponds to the radius of the cluster and the years of the clusters are denoted.

The majority of *S*. Typhi cases (241/284; 85%) originated from communes located within Siem Reap province (Fig 2). The median population density in communes with at least once case of typhoid fever was 119 people/km^2^ (IQR: 60–212), and 70/78 (90%) of communes with a typhoid fever case were classified as rural. Compared to typhoid cases, the non-typhoid fever population controls came from a larger area (243 communes), the median population density of which was lower at 106 people/km^2^ (IQR: 53–210); however, a similar proportion of these communes (220/243; 91%) was also classified as rural (Fig 2).

The estimated median commune level minimum incidence of reported cases of typhoid fever over the study period was 0.62/1,000 children aged <15 years (IQR: 0.37–1.02; range: 0.5–11.36). The reported incidence varied significantly across the 78 communes. Kampong Kleang commune (Soutr Nikom district, Siem Reap) showed the highest incidence of typhoid fever over the study period with 11.36 cases of typhoid fever /1,000 population of children aged <15 years (Fig 2c). This area is renowned for its floating villages and is situated on the edge of Tonle Sap Lake, approximately 35 km southeast of Siem Reap City. The second highest incidence was identified in Kaoh Chiveang commune (Aek Phnum district, Battambang, 33 km southwest of Siem Reap City) with 4.1 cases/1000 people aged <15 years over the study period (Fig 2c). Both of these areas experience heavy flooding when the Tonle Sap Lake expands during the rainy season.

Overall, there was some evidence of positive spatial autocorrelation (case clustering) across the 78 communes that had at least one case of typhoid fever between 2007 and 2014 (Moran’s I = 0.11, *p*<0.056). The magnitude of this autocorrelation varied over time, and was the most significant in 2013 (Moran’s I = 0.19, *p*<0.019) but was non-significant in other years. We were able to identify three significant spatiotemporal clusters associated with high rates of typhoid fever. The first occurred in 2008 toward the west of the study area and had a radius of 23.8 km; this cluster had 1.27 predicted cases and 10 observed cases (relative risk [RR] = 8.17, *p* = 0.002). The second cluster occurred in 2012 in the central northern area and had a radius of 10.8 km, with 1.67 predicted cases and 12 observed cases (RR = 7.47, *p*<0.001). The final cluster occurred in 2013 in the southeastern area and had a radius of 15.5 km, with 0.88 predicted cases and 14 observed cases (RR = 16.8, *p*<0.0001) (Fig 2d).

### The population structure of *Salmonella* Typhi in Siem Reap province, Cambodia

**Fig 3:**
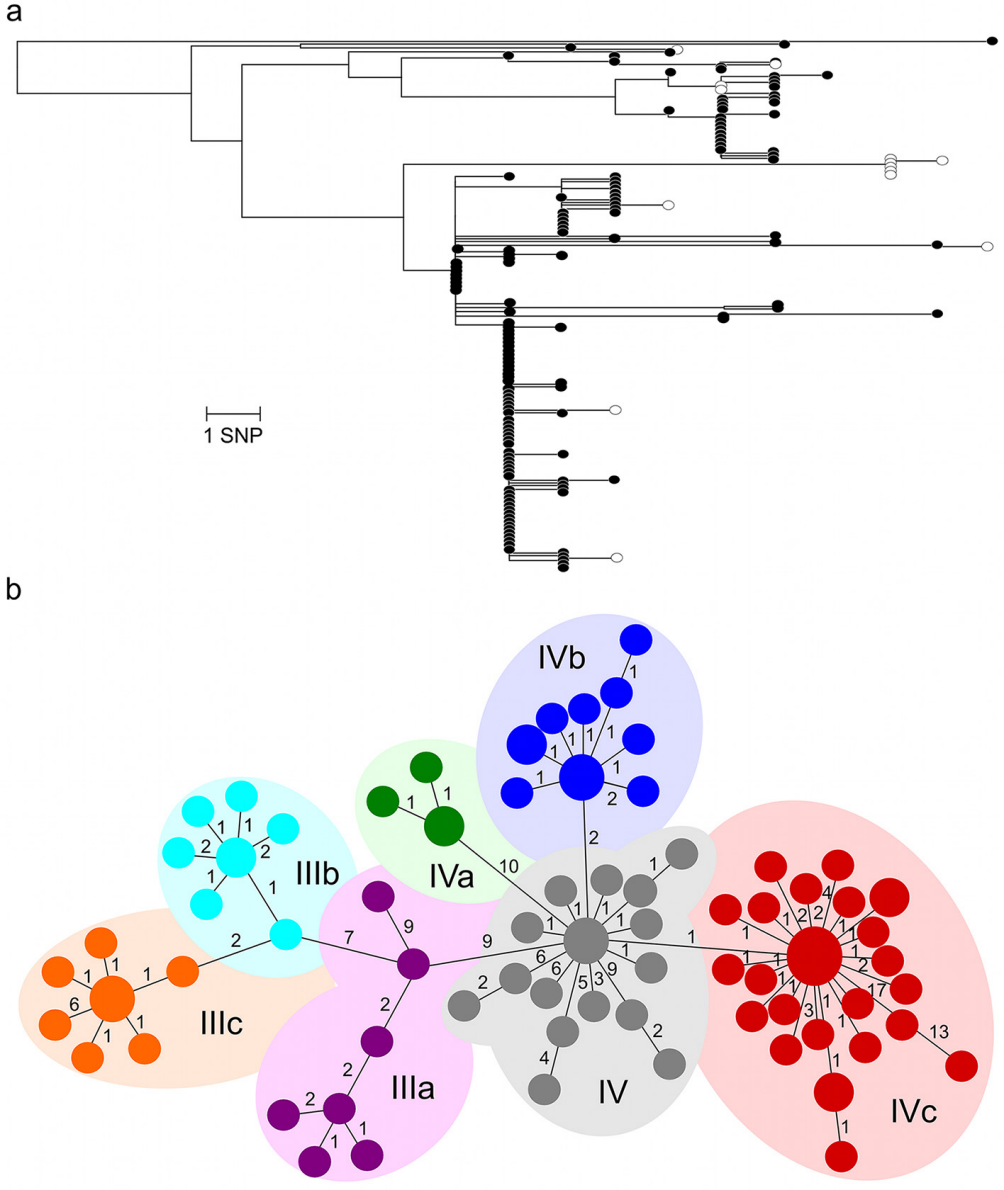
The phylogenetic structure of the H58 lineage of Cambodian *Salmonella* Typhi. a) Maximum likelihood phylogenetic tree of the 203 H58 isolates identified during this project (scale bar denotes SNP differences). The sub-lineages are shown on the major branches. Isolates exhibiting a multi-drug resistance (MDR) phenotype are indicated by black nodes. The tree is midpoint-rooted for the purpose of clarity. Bootstrap values >85% are indicated by an asterisk. b) Minimum spanning tree subdividing H58 lineage III and IV into the various sublineages (IIIa, IIIb, IIIc, IV, IVa, IVb, IVc). The various sublineages are color-coded for reference and the number of each variant is indicated by the cluster size. The number on each of the branches signifies the number of SNPs between each cluster.

The resulting WGS data demonstrated that 97% (203/209) of the sequenced Cambodian isolates could be attributed to haplotype H58. The majority (199/203, 98%) of the H58 isolates exhibited intermediate susceptibility against fluoroquinolones (0.12–0.5 μg/mL) via the common amino acid substitution of serine to phenylalanine at codon 83 (S83F) in the DNA gyrase protein encoded by *gyrA*. There was a strong association between haplotype H58 and an IncHI1 plasmid, which confers an MDR phenotype, with 89% (180/203) of the H58 isolates harboring the common IncHI1 plasmid and the corresponding antimicrobial resistance phenotype. For the six non-H58 isolates, no mutations were observed in the *gyrA* gene, while two (33%) carried the same IncHI1 plasmid as found in the H58 isolates. We identified 188 SNPs across the H58 population and, from a SNP-based phylogeny, identified the circulation of at least four lineages of H58 circulating in the selected area of Cambodia between 2007 and 2012 (Fig 3a). These lineages, designated here as I-IV, differed from each other by as little as three to five SNPs and were phylogenetically well-supported (bootstrap values ≥ 87%). The majority of the H58 isolates fell into lineage IV (152/203, 75%) and lineage III (41/203, 20%).

### The spatiotemporal distribution of *Salmonella* Typhi genotypes

**Fig 4:**
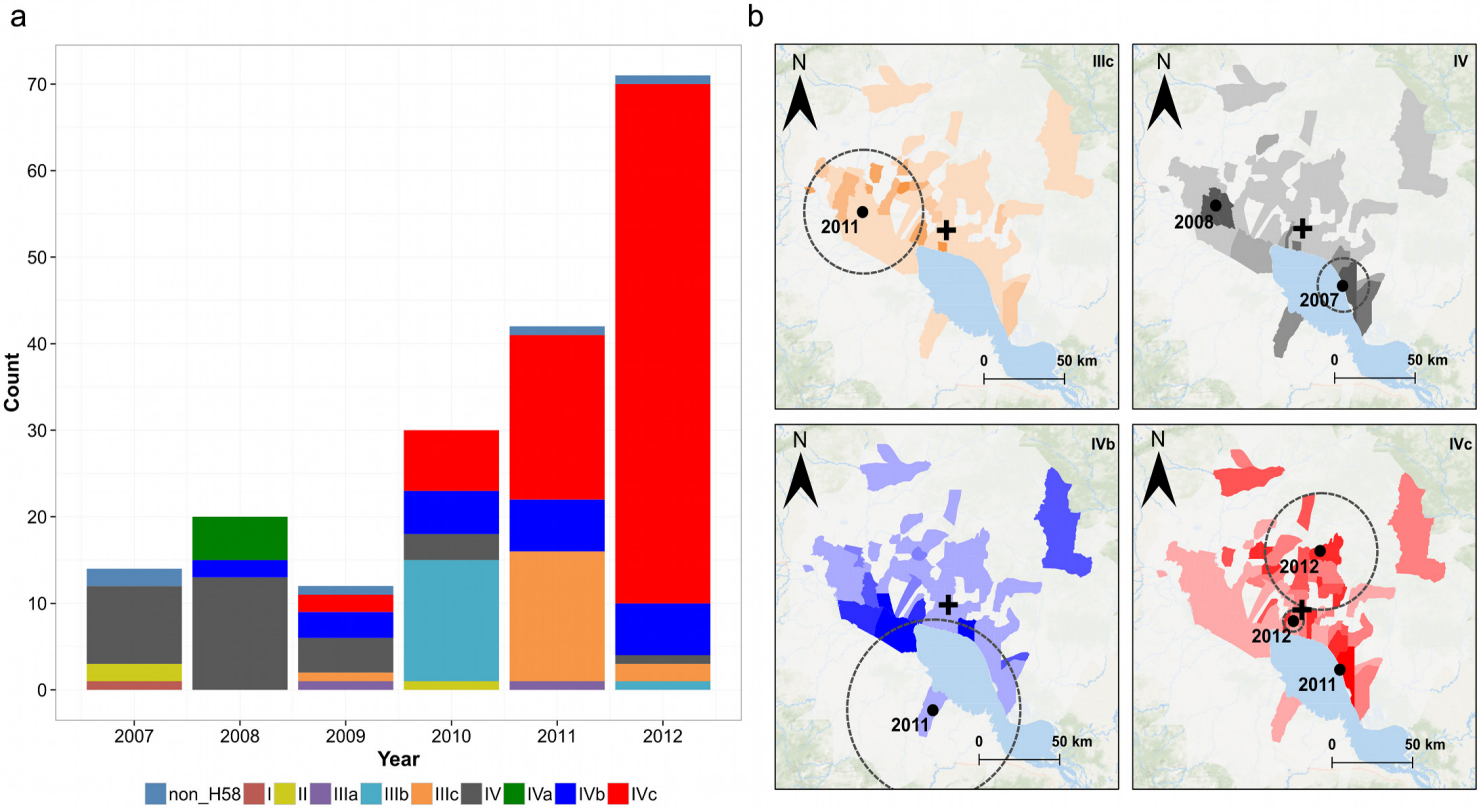
The spatiotemporal distribution of the various *Salmonella* Typhi lineages/sublineages in Siem Reap province, Cambodia. a) Bar chart shows the annual distribution of the various *S*. Typhi lineages/sublineages from 2007 to 2012; sublineages are color-coded as in Fig 3b. b) Maps showing significant spatiotemporal clusters identified for sublineages IIIc, IV, IVb and IVc. The timing of each cluster is shown by the year in black text and the dotted circle represents the radius of the detected cluster. Background colors represent the rate of each sublineage per 1,000 population aged under 15 years. The incidence rates vary between sublineages, ranging from 0 to a maximum of 0.8 (IIIc), 3.12 (IV), 2.56 (IVb) and 5.84 (IVc) 5.84 cases/1,000 population aged under 15 years.

To investigate short-term evolutionary traits within the identified lineages, we constructed a SNP-based minimum spanning tree (Fig 3b). Using these data, we were able to investigate the local population dynamics and detected several clonal clusters emerging from lineage III (IIIa-IIIc) and lineage IV (IVa-IVc); SNPs defining these sublineages are shown in S2 Table. Our data show a complex temporal distribution of *S*. Typhi H58 sublineages circulating in this location between 2007 and 2012 (Fig 4a). The distribution of these various strains was highly dynamic, with strain replacements, potential extinctions and the specific microevolution and expansion of H58-IVc (Fig 4a). In 2011 and 2012, H58-IVc became the dominant genotype, accounting for 44% (18/42) and 85% (61/72) of all *S*. Typhi isolates in these years, respectively.

We next aimed to identify spatiotemporal clustering of the various *S*. Typhi H58 sublineages, and found that IIIc, IV, IVb and IVc all displayed significant evidence of clustering over space and time. Notably, the locations of these clusters were generally different between sublineages, signifying some degree of geographical variation of the circulating *S*. Typhi strains. For example, we identified significant clustering of H58-IIIc in the western part of the study area in 2011 (*p*<0.001, RR: 26.7, radius: 36km) (Fig 4b) and clustering of the emergent H58-IVc strain in both 2011 (Kampong Khleang commune, *p*<0.001, RR: 39.4, radius: <1km) and in two locations in 2012 (smaller cluster, *p* = 0.017, RR: 5.17, radius: 6.2km; larger cluster, *p*<0.001, RR: 5.87, radius: 33.9km).

### Population risk factors for typhoid fever

Finally, we investigated associations between rates of typhoid in children and demographic and sanitation variables at the commune level. We found a number of significant risk factors (e.g. low female education level and collection of drinking water near the household premises) and protective factors (e.g. higher population density, elevation, distance from lake and attendance at school) associated with the rate of typhoid hospitalizations in the univariate analysis (Table 2). However, after controlling for confounders, we found that the distance of the centroid of the commune to the perimeter of the lake was strongly and significantly associated with rate of typhoid cases (10km increase in distance from the lake, incidence rate ratio (IRR): 0.38, 95%CI 0.26–0.55, *p*<0.001) (Table 2). Furthermore, the relative numbers of households within the commune connected to public sewage services and households using a sunken well were also strongly protective, however these associations were reversed through interaction with increasing number of households using wells and distance from the lake, respectively (Table 2). Finally, a high number of households reporting drinking water retrieval from ‘within the household premises’ was also associated with a significant protective effect (log households/1,000 households, IRR: 0.65, 95%CI: 0.49–0.86, *p* = 0.003).

**Table 2:** Regression results highlighting factors associated with typhoid cases.

Commune characteristic	Univariable		Multivariable	
	IRR (95%CI)	p	IRR (95%CI)	P
Population density^	0.81 (0.70–0.95)	0.008		
Elevation, 10m	0.89 (0.81–0.99)	0.026		
Distance to lake, 10km	0.81 (0.74–0.89)	<0.001	0.38 (0.26–0.55)	<0.001
Average household size	1.54 (0.66–3.57)	0.317		
Total attending school/1,000^	0.11 (0.04–0.33)	<0.001		
Female education >25 years /1000 population^				
Primary not completed	2.59 (1.53–4.38)	<0.001		
Primary/Lower secondary	0.94 (0.70–1.25)	0.654		
Secondary or above	0.96 (0.80–1.17)	0.714		
Toilets per 1000 people^				
None	1.24 (0.84–1.79)	0.246		
Sewage	0.96 (0.81–1.15)	0.676	0.44 (0.25–0.80)	0.007
x households with wells			1.19 (1.07–1.32)	0.001
Septic tank	0.89 (0.78–1.02)	0.090		
Pit latrine	0.95 (0.79–1.15)	0.621		
Drinking water, hh/1000 hh^				
Piped	0.87 (0.74–1.03)	0.104		
Tube/pipe well	0.82 (0.73–0.92)	0.001		
Dug well	0.83 (0.64–0.83)	<0.001	0.31 (0.19–0.50)	<0.001
x distance to lake			1.16 (1.09–1.25)	<0.001
Spring/river	1.15 (1.05–1.25)	0.003		
Drinking water location, hh/1000 hh^				
Within premises	0.71 (0.55–0.93)	0.013	0.65 (0.49–0.86)	0.003
Near premises	3.38 (2.24–5.10)	<0.001		
Away premises	0.88 (0.73–1.06)	0.177		

^log of the variable was included; hh: household

## Discussion

In this study we combined conventional epidemiological methods, current genome sequencing tools and geospatial mapping to add insight into the epidemiology of typhoid fever in pediatric patients attending a single healthcare facility in central Cambodia. The majority of recent typhoid fever studies originate from urban locations in low-income countries. This study provides a new perspective into this important community-acquired infection from a predominantly rural setting. The primary finding of this study is that there is a considerable and widespread burden of pediatric typhoid fever in rural Cambodia, thus questioning the dogma that typhoid fever is predominantly geographically restricted to urban populations with poor sanitation systems [9,7]. Our data are consistent with findings from a recent study conducted across sub-Saharan Africa [30]. The Typhoid Surveillance in Africa Programme (TSAP) found a large burden of typhoid fever in younger children and almost equivalent population incidences between urban and rural settings. This distribution was most apparent in West Africa (Burkina Faso and Ghana) and was similarly restricted to children aged less than 15 years [30]. Therefore, we infer that the epidemiology of typhoid fever in Cambodia may be more similar to contemporary observations from sub-Saharan Africa, as opposed to the urban distribution that has commonly been observed across much of Asia [7,31].

The impending availability of Vi-conjugate vaccine raises the question of who should be given this vaccine and when it should be given to obtain maximum benefit in the control of typhoid fever [32,33]. This issue is complicated by a lack of population-based incidence data and a poor understanding of the burden of disease in school and preschool aged children, for whom the conjugated form of the Vi polysaccharide vaccine would be particularly beneficial [34]. Our data indicate a substantial burden of typhoid fever in school and preschool aged children in this area, with a hospital-based incidence (i.e. a minimum population incidence) of 11.36 cases of typhoid fever /1,000 population in children aged <15 years over the study period. The overall burden of typhoid fever in this population is likely to be greater than we have estimated due to poor sensitivity of blood culture and restriction of the study to a single healthcare center. Siem Reap province could be a suitable location in which to trial, or even introduce, the next generation typhoid vaccines in Cambodia that have been tested elsewhere [32]. Further, we suggest that immunizing school-aged children in the period prior to the wet season may provide the most economic and prudent approach for vaccine introduction.

Between 2007 and 2012, we observed a sharp increase in the number of typhoid cases concurrent with an increasing geographic expansion. We also observed that typhoid fever in this population followed a seasonal pattern, suggesting an association with rainfall and potentially with localized flooding and the contamination of water sources. The population-based risk factors support these hypotheses, as living further away from Tonle Sap Lake and access to water within the household were highly protective. Additionally, we found that two communes located next to the lake (Kaoh Chiveang and Kampong Kleang) had the highest incidence of typhoid fever and had large clusters of cases in 2008 and 2013. This case clustering in specific locations warrants further investigation at the household level to understand specific sanitation-associated risk factors and likely exposures to *S*. Typhi in this setting [35]. It appears that access to lake water in some of these communes, such as Kaoh Chiveang, is vital for the household water supply and we hypothesize that the lake water is more prone to localised fecal contamination at specific times throughout the year.

Using targeted SNP-specific PCR, we have previously shown that MDR H58 *S*. Typhi strains dominate in this population [18]. Our WGS investigation confirmed these findings and identified additional diversification in this population. We were able to separate these H58 strains into seven (IIIa, IIIb, IIIc, IV, IVa, IVb, IVc) major sublineages. These discrete groups varied in size and were segregated by only limited numbers of SNPs. We did observe some evidence of expansion of sublineage IVc between 2009 and 2012; this correlated with several spatiotemporal clusters suggesting small disease outbreaks. We currently cannot explain the expansion of this group and our strain selection for sequencing was limited by the availability of strains isolated only up to 2012. Despite some clustering of closely related strains, the overall temporal and spatial distribution of strains was random, with a range of *S*. Typhi H58 sublineages circulating throughout the study period, which is similar to patterns described in urban settings in Asia [14,36].

This study has some limitations. First the data originated from patients attending a single healthcare facility, without the added support of healthcare utilization data. This approach, while cost-effective, induces bias in the spatial and risk factor analyses. Furthermore, while the associations identified in the regression analysis are plausible and provide direction for future investigations, they should be viewed with caution. The population level census data does not allow examination of exposures at an individual or household-level and provides only broad epidemiological evidence. However, the association with distance to the lake and water and sanitation variables suggests these factors should be examined more rigorously in the future with respect to the dynamics of typhoid fever outbreaks. Similarly, the identification and location of the spatiotemporal clusters should be interpreted with some degree of caution. Communes without cases were not included in the cluster analyses due to a lack of data as to whether these regions truly lacked typhoid cases. A dataset with more complete spatial information on presence and absence of typhoid would permit a more reliable analysis.

In conclusion, we find a large burden of typhoid fever in children in rural Cambodia. Our conventional population-based risk factor analysis identified access to water in the household and increasing distance from Tonle Sap Lake as protective against typhoid fever in communes. Spatial mapping and WGS provided additional resolution to investigate these findings and confirmed that proximity to that lake was associated with discrete disease clusters. We confirmed the dominance of MDR H58 *S*. Typhi in this location and found a substantial amount of diversification within this lineage. Our data provide a platform for additional studies in the Cambodian population and suggest that this is a suitable location in which to introduce Vi conjugate vaccines for school children.

## Supporting Information

S1 Table Strain list and accession numbers for organisms used in this study. (XLS)

S2 Table SNPs defining H58 sublineages. (XLS)

S1 Checklist STROBE checklist. (DOC)
